# A surgeon-modified device for the evacuation of diathermy smoke

**DOI:** 10.1308/rcsann.2024.0061

**Published:** 2025-08-29

**Authors:** OD Brown, S Aroori

**Affiliations:** ^1^ University Hospitals Plymouth NHS Trust, UK; ^2^ University of Plymouth, UK

## Background

Diathermy smoke is a recognised occupational hazard, exposing staff to smoke equivalent to between 27 and 30 cigarettes each day.^1^ Consequences of inhalation include acute and chronic inflammatory lung conditions, carcinogenesis and exposure to pathogens including human immunodeficiency virus.^2^ The Health and Safety Executive recommends that, “If exposure to diathermy emissions can't be prevented then it should be adequately controlled … by Local Exhaust Ventilation”.^3^ Commercially available smoke extractors are expensive and costly to maintain. The units used in our hospital, for example, cost £2,040 and require perishable filters. Compatible, single-use diathermy pencils cost on average £16.08. We describe an alternative, simple, cost-effective surgeon-modified device, developed by the senior author.

## Technique

**Figure 1:**
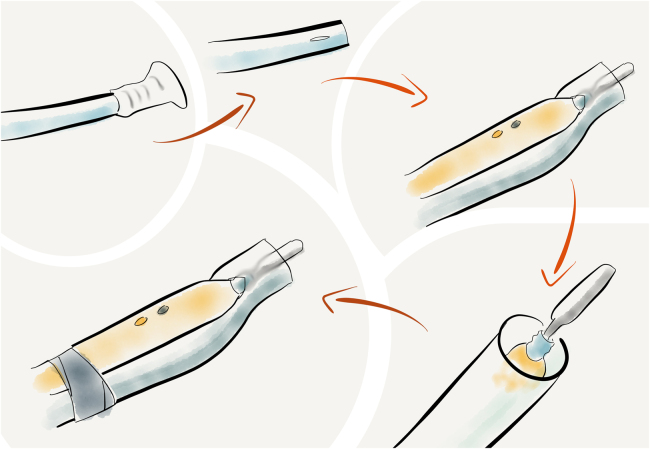
Steps involved in making the surgeon-modified smoke extraction device

This technique requires one length of suction tubing (£0.47), a handheld diathermy pencil (£1.18) and sterile adhesive tape (£0.30). The connector is cut transversely from the suction tubing. A longitudinal slit is made 2cm from the cut end. The diathermy blade is pushed through the slit so that it sits centrally in the opening of the tube. The tube is secured opposite the buttons, using sterile tape (Figure 1). Care is taken to ensure that the tube cannot touch the diathermy blade, to prevent it from melting.

## Discussion

Commercially available smoke extractors are expensive and require dedicated consumables. Where they are unavailable, this potentially useful technique utilises readily available equipment at an incidental cost of £1.95. For safety and efficiency, the theatre should operate two suction devices simultaneously. We have found that the bulk of the modification does not hinder the surgeon.
